# The After-Care of the Disabled Soldier

**Published:** 1918-03-09

**Authors:** A. W. Mayo Robson

**Affiliations:** A.M.S., C.V.O., C.B.


### THE AFTER-CARE OF THE DISABLED SOLDIER.

### By COLONEL A. W. MAYO ROBSON, A.M.S., C.V.O., C.B., F.R.C.S.*

Colonel Mayo Robson said the subject of the after-care of the disabled soldier bristled with difficulties, and it had been considered by the War Office, assisted by a Council of Consultants; it had also been deliberated upon by the Minister of Pensions. There were many cases of men who had been discharged from the Army with conditions which were largely remediable by operation, which procedure, however, they declined. To give an example, the man who worked a lift which the lecturer happened to use was noticed to have wrist-drop. When told it could be cured by operation he said he did not propose to have it done: he was receiving a pension of 28s. a week, on which he could go along comfortably. These men should be made to understand that by getting cured they would not lose their pension, and by undergoing the necessary treatment the total productive power of the nation would be much increased.

For the tubercular cases a considerable number of sanatoria would need to be erected, and there would have to be special centres for neurasthenic cases and shell-shock patients, of which, as Inspector on the Southern Command, he saw large numbers. These did not do well in Command depôts; they should be right away from the sound and the associations of war. Also, in connection with the patients in the many orthopædic hospitals, workshops and training centres were needed, as well as hospitals or asylums for incurable cases, such as cases of paraplegia. He insisted on the need for properly qualified people to be placed in charge of these institutions; those having special knowledge of the treatments and the apparatus; and they should exercise unceasing watchfulness. With very large numbers of war victims a very important function was that of re-educating them to a stage at which they could resume active work.

### Ankylosis: Points to be Noted.

The orthopædic hospitals would have to be continued after the war, to treat and restore stiff joints, fractures with shortening, and nerve cases causing deformities. In cases in which stiffness was inevitable it was most important that the ankylosis should take place with the limb in its most useful position: gunshot injuries through the joints, followed by suppuration, generally were succeeded by ankylosis. In the case of the shoulder, for instance, the arm should be kept abducted at an angle of 50 degrees, with the elbow slightly in front of the body and away from it, so that when it was at right angles and the forearm was supinated, the palm of the hand was towards the face. This enabled the hand to be of service when the arm was moved as a whole from the scapula. With regard to the elbow-joint the position must be always influenced by the patient's calling. In most cases the best functional results were obtained when the angle between the humerus and the forearm was a little greater than a right angle. The forearm should be supinated so that the palm was directed slightly upwards. If both elbows were ankylosed, one should be at an angle of 110° and the other at 70°, so that the movements of one arm could supplement those of the other. All injuries of the wrist should be treated with the wrist dorsi-flexed and with the fingers spread well out. In hip-joint cases ankylosis, if inevitable, should be secured in the position of slight abduction, and the thigh extended and slightly rotated outwards. Knee cases should be fully extended, care being taken to avoid hyper-extension. In injuries of the ankle the foot should be kept at a right angle. He saw many cases in which the toes had been allowed to drop. A valgoid position was specially to be avoided.

### The Handling of Stiff Joints.

One should never try to get a stiff joint moved rapidly, and on no account should an anæsthetic be given for the purpose of moving a stiff joint; this might ultimately lead to permanent damage of the joint. These joints required very careful handling. Massage should be carried out for a few days, and if no reaction followed the arm could be moved a little from the splint, raised, and then replaced, being content with doing only a little each day. If no reaction followed, the procedure might be carried out each day, and the patient encouraged to lift it actively from the splint. When this could be done easily the splint could be bent a little each day, thus enabling further movements to be performed. The underlying principle was one of alternate slight movement and rest. Any reaction must be taken as an indication that further rest was needed. Where injuries involved tissues in the neighbourhood of joints, resulting in suppuration, deep scarring, etc., stiff joints often resulted; and here also no attempt at breaking down should be made, even under an anæsthetic. With regard to injuries occurring at a distance from joints, with inflammatory changes in muscles, no joint which was not the seat of arthritis should ever be allowed to stiffen permanently. When a joint could be moved without disturbing the fragments of a fracture that should be practised. One gentle movement in each direction every other day sufficed.

In reference to stiff fingers all splints for dorsi-flexion of the wrist, especially where fractures or inflammatory changes occurred in the metacarpals and phalanges, should hold the fingers in the position they assume when grasping a cricket-ball, care being taken not to exceed the ordinary metacarpo-phalangeal range, as a stiffening in that posture was impracticable. Massage and passive movement should be very gently practised. Splints should not be applied to the fingers except where they were the actual seat of fracture or active inflammatory changes. In all forearm fractures the hand should be kept supinated; it was often difficult to secure supination. Splints should be removed from the upper extremity at the earliest practicable moment; and in the case of the lower limbs sufficient rest should be given before the patient walks, as the softening of the bones caused by the accident might result in bowing.

* An address given to a meeting N.U.T.N., 46 Marsham Street, Westminster, on February 23, 1918.

